# The CROWN Initiative: Journal Editors Invite Researchers to Develop Core Outcomes in Women’s Health 

**Published:** 2014-11-01

## The Core Outcomes in Women’s Health (CROWN) Initiative

Clinical trials, systematic reviews and guidelines compare beneficial and non-beneficial outcomes following interventions. Often, however, various studies on a particular topic do not address the same outcomes, making it difficult to draw clinically useful conclusions when a group of studies is looked at as a whole (1). This problem was recently thrown into sharp focus by a systematic review of interventions for preterm birth prevention, which found that among 103 randomised trials, no fewer than 72 different outcomes were reported (2). There is a growing recognition among clinical researchers that this variability undermines consistent synthesis of the evidence, and that what is needed is an agreed standardised collection of outcomes-a "core outcomes set"-for all trials in a specific clinical area (1). Recognising that the current inconsistency is a serious hindrance to progress in our specialty, the editors of over 50 journals related to women’s health have come together to support The CROWN (CoRe Outcomes in WomeN’s health) Initiative (Box 1).

Box 1: Aims of The CROWN Initiative

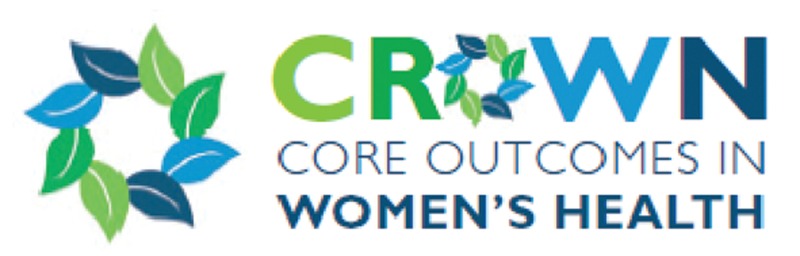

1. Form a consortium among all gynaecologyobstetrics and related journals to promote core outcome sets in all areas of our specialty.
2. Encourage researchers to develop core outcome sets using robust consensus methodology involving multiple stakeholders, including patients.
3. Strongly encourage the reporting of results for core outcome sets.
4. Organise robust peer-review and effective dissemination of manuscripts describing core outcome sets.
5. Facilitate embedding of core outcome sets in research practice, working closely with researchers, reviewers, funders and guideline makers.

www.crown-initiative.org

Development of consensus is required around a set of well-defined, relevant and feasible outcomes for all trials concerning particular obstetric and gynaecologic health conditions, such as preterm birth, incontinence, infertility and menstrual problems. With so many subspecialties involved, this is no easy task. Duplication of effort can be avoided by working with the Core Outcome Measures in Effectiveness Trials (COMET) Initiative, which is working towards core data sets for all medical specialties (3). Production of trustworthy core outcome sets will require engagement with patients, healthcare professionals, researchers, industry and regulators, and the employment of scientifically robust consensus methods (1). The data for these core outcome sets, once agreed upon, should be collected in trials and reported in publications as standard practice in the future.

Journal editors now invite researchers to take the lead in beginning this work. What will we do as editors to support them and their colleagues? First, we are drawing wide attention to The CROWN Initiative by publishing this editorial in the journals listed below. We shall ensure that the global research community, which includes our many reviewers, is aware of the need for core outcome sets. Submissions which describe development of core outcome sets, if deemed acceptable after peer review, will be effectively disseminated.

Our collaboration is not for enforcing harmony at the expense of innovation. To quote from the COMET home page (www.comet-initiative.org): "The existence or use of a core outcome set does not imply that outcomes in a particular trial should be restricted to those in the relevant core outcome set. Rather, there is an expectation that the core outcomes will be collected and reported, making it easier for the results of trials to be compared, contrasted and combined as appropriate; while researchers continue to explore other outcomes as well." We also expect that as new or superior ways of capturing outcomes emerge, core outcome sets will themselves need updating.

Producing, disseminating and implementing core outcome sets will ensure that critical and important outcomes with good measurement properties are incorporated and reported. We believe this is the next important step in advancing the usefulness of research, in informing readers, including guideline and policy developers, who are involved in decision-making, and in improving evidence-based practice.

## Notes

Reproduced from ***The Core Outcomes in Women’s Health (CROWN) Initiative*** with permission from the Royal College of Obstetricians and Gynaecologists and John Wiley & Sons Ltd.

## Appendix

The CROWN Initiative includes the following journals, in alphabetical order (correct on 13^th^ May 2014, up to date list available at www.crown-initiative.org):

1. Acta Obstetricia et Gynecologica Scandinavica
2. American Journal of Obstetrics & Gynecology
3. American Journal of Perinatology
4. Archives of Gynecology and Obstetrics
5. Australian and New Zealand Journal of Obstetrics and Gynaecology
6. Best Practice & Research: Clinical Obstetrics & Gynaecology
7. Birth: Issues in Perinatal Care
8. BJOG: An International Journal of Obstetrics and Gynaecology
9. BMC Pregnancy and Childbirth
10. BMC Women’s Health
11. Climacteric
12. Clinical Obstetrics and Gynecology
13. Clinics in Perinatology
14. Cochrane Menstrual Disorders and Subfertility Group
15. Cochrane Pregnancy and Childbirth Group
16. Contraception
17. Current Opinion in Obstetrics and Gynecology
18. European Journal of Obstetrics & Gynecology and Reproductive Biology
19. Fertility and Sterility
20. Fetal Diagnosis and Therapy
21. Ginekologia Polska
22. Gynecological Surgery
23. Gynecologic Oncology
24. Gynecologic Oncology Reports
25. Human Fertility
26. Human Reproduction
27. Human Reproduction Update
28. Hypertension in Pregnancy
29. International Journal of Fertility and Sterility
30. International Breastfeeding Journal
31. International Journal of Gynecology & Obstetrics
32. International Urogynecology Journal
33. Journal of Family Planning and Reproductive Health Care
34. Journal of Gynecologic Oncology
35. Journal of Lower Genital Tract Disease
36. Journal of Midwifery & Women’s Health
37. Journal of Obstetrics & Gynaecology
38. Journal of Obstetrics and Gynaecology Canada
39. Journal of Obstetric, Gynecologic & Neonatal Nursing
40. Journal of Perinatal & Neonatal Nursing
41. Journal of Perinatal Medicine
42. Maturitas
43. MCN The American Journal of Maternal Child Nursing
44. Menopause Review (Przegl¹d Menopauzalny)
45. Menopause: The Journal of The North American Menopause Society
46. Neurourology and Urodynamics
47. Obstetrics & Gynecology
48. Paediatric and Perinatal Epidemiology
49. Placenta
50. Prenatal Diagnosis
51. Reproductive Health
52. The Breast Journal
53. The European Journal of Contraception and Reproductive Health Care
54. The Obstetrician & Gynaecologist (TOG)
55. Twin Research and Human Genetics
56. Ultrasound in Obstetrics & Gynecology
